# Designer Leptin Receptor Antagonist Allo-aca Inhibits VEGF Effects in Ophthalmic Neoangiogenesis Models

**DOI:** 10.3389/fmolb.2016.00067

**Published:** 2016-10-13

**Authors:** Roberta Coroniti, Rafal Farjo, Didier J. Nuno, Laszlo Otvos, Laura Scolaro, Eva Surmacz

**Affiliations:** ^1^Sbarro Institute for Cancer Research and Molecular Medicine, Temple UniversityPhiladelphia, PA, USA; ^2^Department of Biology, Temple UniversityPhiladelphia, PA, USA; ^3^EyeCROOklahoma, OK, USA

**Keywords:** leptin, ObR antagonist, peptide drug, VEGF, ocular neoangiogenesis

## Abstract

Experimental and clinical data suggest that pro-angiogenic, pro-inflammatory and mitogenic cytokine leptin can be implicated in ocular neovascularization and other eye pathologies. At least in part, leptin action appears to be mediated through functional interplay with vascular endothelial growth factor (VEGF). VEGF is a potent regulator of neoangiogenesis and vascular leakage with a proven role in conditions such as proliferative diabetic retinopathy, age-related macular degeneration and diabetic macular edema. Accordingly, drugs targeting VEGF are becoming mainstream treatments for these diseases. The crosstalk between leptin and VEGF has been noted in different tissues, but its involvement in the development of eye pathologies is unclear. Leptin is coexpressed with VEGF during ocular neovascularization and can potentiate VEGF synthesis and angiogenic function. However, whether or not VEGF regulates leptin expression or signaling has never been studied. Consequently, we addressed this aspect of leptin/VEGF crosstalk in ocular models, focusing on therapeutic exploration of underlying mechanisms. Here we show, for the first time, that in retinal (RF/6A) and corneal (BCE) endothelial cells, VEGF (100 ng/mL, 24 h) stimulated leptin mRNA synthesis by 70 and 30%, respectively, and protein expression by 56 and 28%, respectively. In parallel, VEGF induced RF/6A and BCE cell growth by 33 and 20%, respectively. In addition, VEGF upregulated chemotaxis and chemokinesis in retinal cells by ~40%. VEGF-dependent proliferation and migration were significantly reduced in the presence of the leptin receptor antagonist, Allo-aca, at 100–250 nmol/L concentrations. Furthermore, Allo-aca suppressed VEGF-dependent long-term (24 h), but not acute (15 min) stimulation of the Akt and ERK1/2 signaling pathways. The efficacy of Allo-aca was validated in the rat laser-induced choroidal neovascularization model where the compound (5 μg/eye) significantly reduced pathological vascularization with the efficacy similar to that of a standard treatment (anti-VEGF antibody, 1 μg/eye). Cumulatively, our results suggest that chronic exposure to VEGF upregulates leptin expression and function. As leptin can in turn activate VEGF, the increased abundance of both cytokines could amplify pro-angiogenic and pro-inflammatory environement in the eye. Thus, combined therapies targeting ObR and VEGF should be considered in the treatment of ocular diseases.

## Introduction

Leptin, a pluripotentcytokine produced in the adipose tissue, has been first discovered as a hormone regulating energy balance and appetite via hypothalamic signals (Wauters et al., 2000; Scolaro et al., 2010). In addition to its metabolic functions in the CNS, leptin is known to regulate multiple physiological and pathological processes, e.g., immune responses, hematopoiesis, bone remodeling, cardiovascular function, normal and neoplastic cell growth (Surmacz, 2013; Surmacz and Otvos, 2015; Upadhyay et al., 2015; Meek and Morton, 2016; Naylor and Petri, 2016). Although adipocytes are the main source of leptin, the hormone can be produced by different types of cells and in different organs (Scolaro et al., 2010; Sweeney, 2010; Surmacz, 2013).

This study focuses on the angiogenic function of leptin and its involvement in ocular neovascularization. The leptin receptor (ObR) is expressed in vascular endothelial cells and studies *in vitro* demonstrated that leptin can induce angiogenic differentiation as well as proliferation and migration of endothelial cells, including cells of ophthalmic origin (Bouloumié et al., 1998; Sierra-Honigmann et al., 1998; Cao et al., 2001; Park et al., 2001; Anagnostoulis et al., 2008; Ferla et al., 2011; Garonna et al., 2011; Scolaro et al., 2013; Parrino et al., 2014; Adya et al., 2015). In mouse models, transgenic overexpression of the leptin gene (*ob*) potentiated ischemia-induced retinal neovascularization, while leptin deficiency due to *ob* inactivation, significantly reduced ocular angiogenesis, proving again the role of this cytokine in neovascularization (Suganami et al., 2004). Similarly, leptin was not able to induce neovascularization in corneas of *fa/fa* Zucker rats that lack functional ObR, underlying the importance of leptin signaling in this process (Sierra-Honigmann et al., 1998).

We have recently demonstrated that leptin is a potent mitogenic and angiogenic factor in retinal and corneal endothelial cells (Scolaro et al., 2013; Parrino et al., 2014). We have described that these leptin functions are associated with the modulation of the activity or expression of several signaling molecules involved in proliferation, inflammatory activity and angiogenesis, including the transcription factor STAT3, common kinases Ras, ERK1/2 and Akt, pro-inflammatory mediators and regulators COX2 and NF-κB. Furthermore, we have found that leptin can upregulate its own mRNA and protein expression in retinal and corneal cells, suggesting the existence of leptin autocrine circuits in the eye. We have validated leptin involvement in the above processes using a selective and highly efficacious ObR antagonist Allo-aca, a leptin peptidomimetic that blocks ObR activation and biological activity at low-mid nmol//L concentrations *in vitro* in different cell types (Scolaro et al., 2013; Parrino et al., 2014).

The mechanisms of leptin expression in the eye are still under investigation. Previous studies have shown that eye injury, ischemic or hyperglycemic conditions can increase leptin expression (Suganami et al., 2004; Sun et al., 2010, 2011). We have recently reported that hyperglycemia can induce leptin mRNA and protein expression in retinal endothelial cells and that this process is associated with increased angiogenesis, cell growth and migration. These effects can be partially reversed by ObR antagonist Allo-aca, implicating leptin signaling in the pathological processes caused by high glucose levels (Parrino et al., 2014).

Adding to the bulk of experimental data, some recent clinical reports suggest that leptin can be involved in eye pathologies. For instance, in patients with proliferative diabetic retinopathy (PDR) or retinal detachment (RD), intravitreous leptin levels were significantly elevated compared with that in patients with other ocular diseases (Gariano et al., 2000; Kovacs et al., 2015). In addition, the study suggested that locally produced leptin, not simply leptin derived from circulation, could be involved in the pathogenesis of PDR and RD (Gariano et al., 2000). Similarly, a small study confirmed higher vitreous leptin levels in PDR relative to other retinopathies (Maberley et al., 2006). Whether leptin is causally related to the progression of DR is still under investigation.

An important aspect of leptin's role in the regulation of key processes implicated in eye diseases is its functional connection with vascular endothelial growth factor (VEGF), a major regulator of neoangiogenesis and vascular leakage with a proven role in ocular pathologies such as PDR, age-related macular degeneration (AMD) and diabetic macular edema (DME) (Miller, 2016). Notably, experimental evidence suggests that leptin can induce and amplify VEGF expression and signaling. For instance, in tetrandrine-induced corneal neovascularization model, leptin is found coexpressed with VEGF (Sun et al., 2011). In ischemia-induced neovascularization, leptin can potentiate vessel formation through induction of VEGF expression (Suganami et al., 2004). In endothelial HUVEC cells, leptin stimulats angiogenesis simultaneously with upregulation of VEGF expression (Bouloumié et al., 1998; Sierra-Honigmann et al., 1998). Furthermore, in HUVEC cells, leptin-mediated angiogenesis and intracellular signaling through the p38 MAPK/Akt/COX-2 pathway is partially reduced with a VEGFR inhibitor, implicating this receptor in leptin response (Garonna et al., 2011). In syngeneic mammary cancer models, the inhibition of leptin signaling significantly reduces the levels of VEGF and its receptor VEGFR2 (Newman and Gonzalez-Perez, 2014). The codependence of VEGF and leptin expression has been also noted in *ob/ob* mice where lack of functional leptin is associated with increased circulating concentrations of VEGF-A and leptin replacement normalizes VEGF-A levels in this model. In addition, leptin regulation of VEGF-A expression has been demonstrated in obese subjects before and after weight loss (Gómez-Ambrosi et al., 2010).

However, whether or not leptin/VEGF crosstalk is a bilateral relationship, i.e., if VEGF can influence leptin expression and function, has never been studied. Consequently, this aspect of leptin/VEGF crosstalk and the potential of therapeutic exploration of the underlying mechanism is the subject of the present paper.

At present, several biologic drugs targeting VEGF and/or its receptor have been approved for ophthalmology use (van der Giet et al., 2015; Miller, 2016). However, new treatments, perhaps with broader therapeutic spectrum, are needed to decrease adverse effects and/or complement anti-VEGF drugs (Tang and Kern, 2011; Truong et al., 2011; Chen et al., 2013; van der Giet et al., 2015). In this context, targeting leptin, a known pro-angiogenic and pro-inflammatory factor whose function is intimately related to VEGF, could prove provide an attractive targeted therapy for pathological neovascularization in the eye (Cheung et al., 2010).

## Materials and methods

### Reagents

The ObR antagonist, Allo-aca, a short leptin-based peptidomimetic (H-alloThr-Glu-Nva-Val-Ala-Leu-Ser-Arg-Aca-NH_2_) was used to inhibit ObR signaling and function. The process of Allo-aca design, development and efficacy *in vitro*, including the ophthalmic models, and *in vivo* has been reported by us before (Otvos et al., 2011a,b; Scolaro et al., 2013; Parrino et al., 2014). VEGF (human recombinant, VEGF 165) was purchased from Gibco Life Technologies (Grand Island, NY).

### Cell lines and growth conditions

The cellular assays were performed using monkey endothelial retinal cells (RF/6A) and bovine endothelial corneal cells (BCE). The cells were purchased and cultured as recommended by the supplier (American Type Culture Collection, Rockville, MD, USA) and describe by us in detail previously (Scolaro et al., 2013). Cell culture reagents and media were purchased from Cellgro (Herndon, VA, USA). Before treatments, the cells were synchronized in serum-free medium (SFM) containing 10 μM FeSO_4_ 0.5% bovine serum albumin, 1% FBS, 1% Pen/Strep. We have shown before that RF/6A and BCE ocular endothelial cell lines express ObR and respond to leptin with the activation of various biological functions, i.e., growth, signaling, angiogenesis, migration (Scolaro et al., 2013; Parrino et al., 2014).

### Proliferation assay

The cells (5–7th passage) were plated in 24-well plates at concentrations 5–8 × 10^4^ and 1–1.5 × 10^5^ cells/well for RF/6A and BCE cells, respectively. At semi-confluence, the cells were shifted to SFM for 24 h and then treated with 50–250 ng/mL of VEGF for 24 h, without 100–250 nmol/L Allo-aca. All assays were done in triplicate and repeated 3 times. Cell numbers were determined by direct counting as describe by us previously (Scolaro et al., 2013). The percentage decrease/increase in cell number vs. control SFM was calculated and expressed as mean ± standard deviation (SD).

### Quantitative real time PCR (qRT-PCR)

Leptin mRNA was detected by qRT-PCR as described in detail previously (Scolaro et al., 2013). Briefly, RF/6A and BCE cells at semi-confluence were placed in SFM for 24 h, pretreated or not with 250 nmol/L Allo-aca for 1 h, and then treated with 100 ng/mL VEGF for 6 and 24 h. RNA was isolated from cultures using Trizol Reagent (Life Technologies, Grand Island, NY) and 4 μg of RNA was reverse transcribed using the High-Capacity cDNA Kit (Life Technologies). The RT products were used to amplify leptin sequences using TaqMan probes Bt03211909_m1 for bovine leptin (Gene ID: 280836) and Rh02788316_m1 for monkey leptin (Gene ID: 698728) (Life Technologies). For normalization, parallel reactions were run on each sample for β-actin using a TaqMan probe (Life Technologies). The levels of leptin mRNA relative to β-actin mRNA were determined using a comparative CT method (Life Technologies). All reactions were done in triplicate and an average CT value (±SD) for all RNAs was calculated. The individual experiments were repeated at least 3 times.

### Wound-healing (scratch) assay

Directional cell migration *in vitro* was assessed using a wound-healing assay. Linear scratches (3 per plate) were produced in 100% confluent cultures of RF/6A cells using a 200 μL tip. The cultures were then shifted to SFM, SFM containing 100 ng/mL VEGF, or SFM with 100 ng/mL VEGF plus 100–250 nmol/L Allo-aca for 24 h. Wound dimensions (at least 6 fields/experimental condition) were recorded with Olympus 1 × 81 phase-contrast microscope at 2.0x magnification and images were acquired using Metamorph 7.5 program. The scratch areas were quantified using the Adobe Acrobat Pro program and the areas expressed in arbitrary units (AU).

### Transwell migration assays

The effects of VEGF on chemotactic properties of RF/6A cells were studied using Transwell inserts (8.0 μm pore size) (Corning, Tewksbury, MA). The cells (5–6th passage) were plated at concentrations 5 × 10^4^ cells/well and allowed to migrate through the membrane for 24 h. Then, non-migrated cells in the upper chamber were removed and the cells that migrated across the membranes were stained with Giemsa for 20 min and counted. To test chemotactic effects of VEGF on RF/6A cells, 100 ng/mL VEGF was added to the lower chamber only. The involvement of VEGF-dependent chemokinesis was assessed using 100 or 250 nmol/L Allo-aca. Each migration assay was done in triplicate and repeated at least 3 times and the mean number of migrated cells ± SD was determined.

### Immunofluorescence

Leptin protein was detected in RF/6A and BCE cells by immunofluorescence (IF), as described by us before (Bartella et al., 2008; Cascio et al., 2008; Scolaro et al., 2013). In short, 1 × 10^5^ cells were plated on glass coverslips in normal growth medium. After 24 h, the cells were synchronized in SFM for 24 h and then treated either with 100 ng/mL VEGF in the absence or presence of 100 or 250 nmol/L Allo-aca for 24 h. Next, the cells were washed with PBS, fixed in methanol, and permeabilized in 0.2 Triton X-100%. Leptin expression was detected using pAb A-20 (1:25 dilution; 2 h) and goat anti-rabbit IgG-FITC (1:1000 plus 1.5% blocking goat serum; 1 h). In control experiments, primary Abs were replaced by non-immune serum. To visualize cell nuclei, the coverslips were mounted with UltraCruz Mounting Medium containing DAPI (5 μg/mL of 4′,6-diamidino-2-phenylindole). The expression of leptin was detected using Olympus 1 × 81 phase-contrast microscope at 3.2x magnification. The percentage of positive cells was determined in 10 visual fields. All reagents were purchased from Santa Cruz Biotechnology (Dallas, TX).

### Intracellular signaling

The effects of Allo-aca on VEGF-induced signaling in RF/6A cells were tested by Western immunoblotting (WB). In brief, cells were synchronized in SFM for 24 h and then treated with 100 ng/mL VEGF in the presence or absence of 250 nmol/L Allo-aca for 24 h or were left untreated. Next, total cellular proteins were obtained and the expression and activation of signaling molecules was evaluated with specific antibodies (Abs) as described previously (Scolaro et al., 2013). The following primary Abs from Cell Signaling Technology (Danvers, MA) were used: phospho-Akt, Akt Ser473 pAb, 1:500; total Akt, Akt pAb, 1:1000; phospho-STAT3, STAT3 Tyr705, D3A7 mAb, 1:500; total STAT3, STAT3 79D7 mAb, 1:500; phospho-ERK1/2, p44/42 mitogen-activated protein kinase (MAPK; ERK1/2) pAb Thr202/Tyr204, 1:1000; total ERK1/2, p44/42 MAPK pAb, 1:1000. The experiments were repeated at least 3 times.

### Densitometry evaluation of protein expression

The intensity of bands corresponding to studied proteins was measured in all WB as described before using Image J program (National Institutes of Health; Scolaro et al., 2013). The modifications in protein expression/phosphorylation were evaluated as decrease/increase vs. SFM (% ± SD), differences with *p* ≤ 0.05 were considered significant.

### Laser-induced choroidal neovascularization (CNV) assays

All animal experiments conformed to the ARVO Statement for the Use of Animals in Ophthalmic and Vision Research. A 22-day study was conducted in female 6 week old Brown Norway rats to determine the antiangiogenic/vascular disrupting effects of the leptin receptor antagonist, Allo-aca, in a laser induced model of CNV. The animals were divided into 3 separate treatment groups of 6 animals per group. On day 1, laser treatments were performed on all groups using a 520 nmol/L thermal laser to generate a total of three lesions per eye. On day 3, group 1 received bilateral intravitreal (ivt) injections of the vehicle, group 2 (positive control) received bilateral ivt injections of an anti-VEGF antibody (R&D Systems, AF564) at 1 μg/eye, and group 3 (test) received bilateral intravitreal injections of Allo-aca at 5 μg/eye. Intravitreal administration of Allo-aca was well tolerated by the rats and no adverse events were observed. On Day 22 (3-weeks post-laser treatment), fluorescein angiography was performed and lesion size area was determined following hand tracing of the lesions using image analysis software ImageJ. The experiments were performed under animal protocol 11-156-H approved by the IACUC committee of the University of Oklahoma Health Sciences Center.

### Statistical analysis

The results of *in vitro* and CNV experiments were analyzed by a two-tailed distribution paired Student's *t*-test; *p* ≤ 0.05 were considered statistically significant.

## Results

### VEGF induces leptin mRNA and protein expression in ocular endothelial cells. Allo-aca reduces these effects

The effects of VEGF on leptin expression were assessed in ocular endothelial cell models RF/6A and BCE. In both cell lines, VEGF was tested at 100 ng/mL concentrations for 24 h. The treatment significantly induced leptin mRNA expression in both cell lines (Table 1). VEGF upregulation of leptin mRNA was more pronounced in RF/6A cells (~1.7-fold) vs. BCE cells (~1.3-fold).

**Table 1:** **VEGF induces leptin mRNA expression in BCE and RF6A cells**.

	**BCE Leptin mRNA levels (fold ± SD over SFM)**	**RF/6A Leptin mRNA Expression (fold ± SD over SFM)**
SFM	1.0	1.0
VEGF	1.3 ± 0.2^*^	1.7 ± 0.1^*^
VEGF + Allo-aca	0.9 ± 0.1^#^	0.6 ± 0.2^#^

Leptin mRNA levels in cells treated with 100 ng/mL VEGF for 24 h were measured by QRT-PCR as described in Materials and Methods. Statistically significant differences (p ≤ 0.05) vs. SFM are marked with ^*^ and vs. VEGF with ^#^.

**Figure 1:**
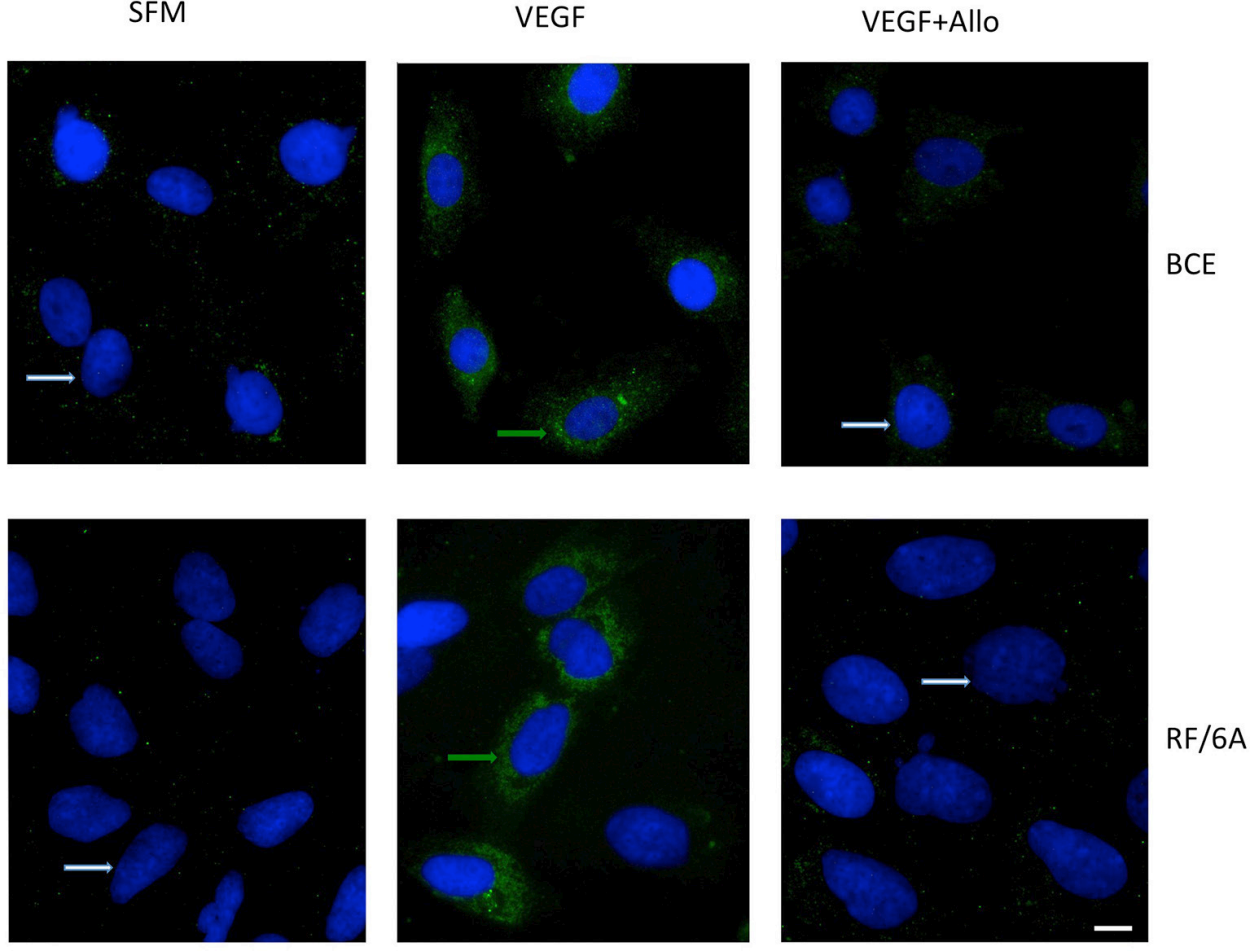
**VEGF induces leptin protein expression in BCE and RF6A cells**. Cells were synchronized in SFM and stimulated with 100 ng/mL VEGF for 24 h in the presence or absence of 250 nmol/L Allo-aca (Allo). Control cells were left untreated in SFM. The expression of leptin protein (green immunofluorescence) was detected with specific Abs while cell nuclei (blue fluorescence) were detected with DAPI, as described in Materials and Methods. The bar represents 10 μm.

Similarly, exposure to VEGF for 24 h significantly increased the number of cells with well detectable expression of the leptin protein. In both cell lines, the level of leptin-positive cells under SFM conditions was below 1%, while upon VEGF treatment, 56 ± 5% of RF/6A cells and 28 ± 3% of BCE cells displayed leptin expression (Figure 1). The upregulation of leptin expression in both cell lines by VEGF was statistically significant.

As leptin is known to stimulate its own expression, we probed if the inhibition of leptin signaling can decrease the above VEGF effects. To this end, we employed a peptide antagonist of the leptin receptor, Allo-aca, that has been shown to block leptin signaling and action in numerous *in vitro* and *in vivo* models (Otvos et al., 2011a,b; Scolaro et al., 2013; Parrino et al., 2014). In the present work, Allo-aca at 250 nmol/L reduced VEGF-dependent leptin mRNA expression in both cell lines below base levels (Table 1). Similarly, addition of Allo-aca at 250 nmol/L to VEGF treatment reduced the number of leptin-positive cells in RF/6A and BCE cultures by 75 ± 4 and 80 ± 5%, respectively (Figure 1).

### ObR antagonist Allo-aca inhibits VEGF mitogenic effects

In addition to strong pro-angiogenic activities, VEGF is known to induce mitogenesis in different endothelial cell models (Lu et al., 2010). Similarly, leptin can increase cell growth in BCE and RF/6A cells, as previously demonstrated by us (Scolaro et al., 2013). Because VEGF increased leptin expression in these cell models, we speculated that at least part of VEGF mitogenic action is mediated through the leptin/ObR axis. To test this hypothesis, we examined if VEGF-mediated growth could be reduced in the presence of the ObR antagonist, Allo-aca.

First, we found that VEGF at 50–250 ng/mL induced proliferation in BCE and RF/6A cells. In both cell lines, the best growth response was observed with VEGF used at 100 ng/mL (Table 2). In the presence of Allo-aca at 100 or 250 nmol/L, VEGF-induced proliferation was either reduced to base or below base levels (Table 2), suggesting that leptin pathways are implicated in VEGF response.

**Table 2:** **VEGF induces cell growth in BCE and RF/6A cells**.

**Treatment**	**BCE Growth response (% ± SD over SFM)**	**RF/6A Growth response (% ± SD over SFM)**
VEGF 50	9.0 ± 0.9^*^	7.1 ± 0.2^*^
VEGF 100	20.4 ± 1.7^*^	33.1 ± 1.9^*^
VEGF 250	16.2 ± 1.8^*^	29.3 ± 1.9^*^
VEGF 100 + Allo-aca 100	−4.0 ± 0.0^*^	4.0 ± 0.1^*^
VEGF 100 + Allo-aca 250	−18.0 ± 1.2^*^	−4.1 ± 1.3

Allo-aca inhibits VEGF mitogenic effects. Proliferation assays were carried out as described in Materials and Methods. Increase of cell number (%) over that in SFM is shown. VEGF was used at 50–250 ng/mL; Allo-aca was used at 100 and 250 nmol/L. Statistically significant differences (p ≤ 0.05) vs. SFM are marked with asterisk.

### Allo-aca inhibits VEGF-induced chemotaxis and chemokinesis in RF/6A retinal endothelial cells

VEGF as well as leptin are well recognized as regulators of chemotaxis and chemokinesis–processes that are intimately involved in angiogenic differentiation. To assess if VEGF effects on chemotaxis and chemokinesis are mediated indirectly through ObR, we used RF/6A cells, characterized by robust migratory abilities *in vitro*. The migration of RF/6A cells was measured in wound-healing and Transwell assays.

In wound-healing assay, basal cell migration was observed even in SFM at 24 h after cell plating, likely due to the activity of autocrine pro-migratory factors (e.g., leptin) produced by RF/6A cells (Scolaro et al., 2013).

**Figure 2:**
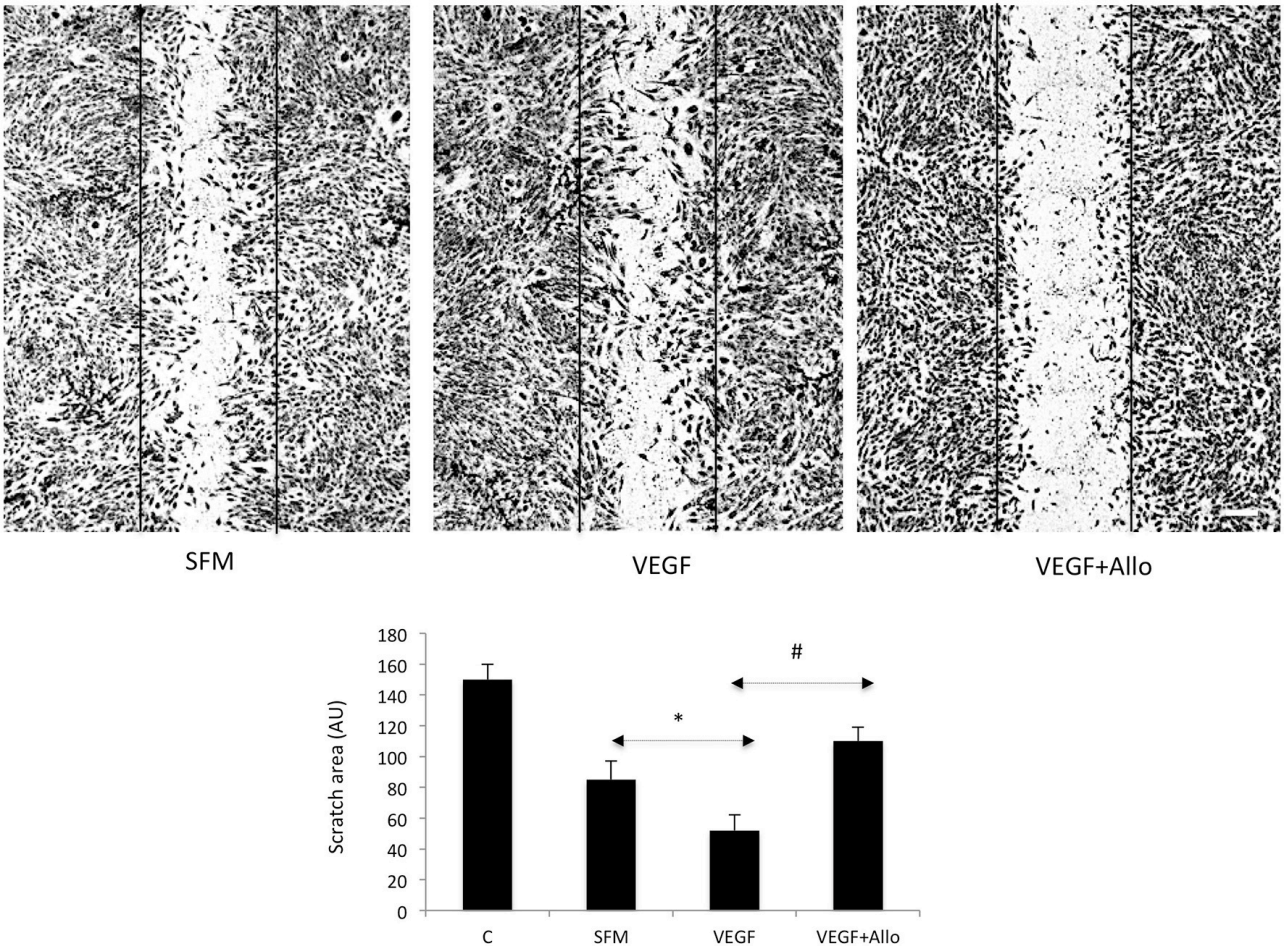
**Allo-aca inhibits VEGF-induced chemokinesis in RF6A cells**. The scratch assays were preformed as described in Materials and Methods. Immediately after wounding, the cells were photographed (control, C) and then placed for 24 h in either SFM or incubated with 100 ng/mL VEGF in the presence or absence of 250 nmol/L Allo-aca (Allo). The scratch areas before and after treatments were measured as described in Materials and Methods and the data are shown in the graph. Statistically significant changes (*p* ≤ 0.05) are marked (SFM vs. VEGF ^*^; VEGF vs. VEGF+Allo-aca ^#^). The initial scratch boundaries (C) have been marked in the picture. The bar represents 250 μm.

Addition of VEGF at 100 ng/mL further stimulated cell motility, reflected by significantly reduced (~40%) scratch area compared with that under untreated SFM conditions (*p* ≤ 0.05) (Figure 2). The effects of VEGF were totally abolished (*p* ≤ 0.05) in the presence of 250 nmol/L Allo-aca (Figure 2).

In the Transwell assay, VEGF at 100 mg/mL induced directional migration of ~37% of RF/6A cells. Addition of Allo-aca at 100 nmol/L partially (~46%) reduced VEGF effects, while Allo-aca at 250 nmol/L blocked VEGF-directed migration by ~92% (Table 3).

**Table 3:** **Allo-aca inhibits VEGF-induced chemotaxis in RF/6A cells**.

**Treatment**	**Migrating cells (% ± SD over SFM)**
SFM	0.0
VEGF	37.6 ± 7.0^*^
VEGF + Allo-aca 100	20.4 ± 2.3^*^
VEGF + Allo-aca 250	3.4 ± 0.8

Chemotaxis induced by VEGF was measured in Boyden chamber assays as described in Materials and Methods. VEGF was used at 100 ng/mL; Allo-aca was used at 100 and 250 nmol/L. The number of migrating cells in SFM is taken as 0%. Statistically significant differences (p ≤ 0.05) vs. SFM are marked with asterisk.

### Allo-aca inhibits several VEGF-induced intracellular signals in RF/6A retinal endothelial cells

**Figure 3:**
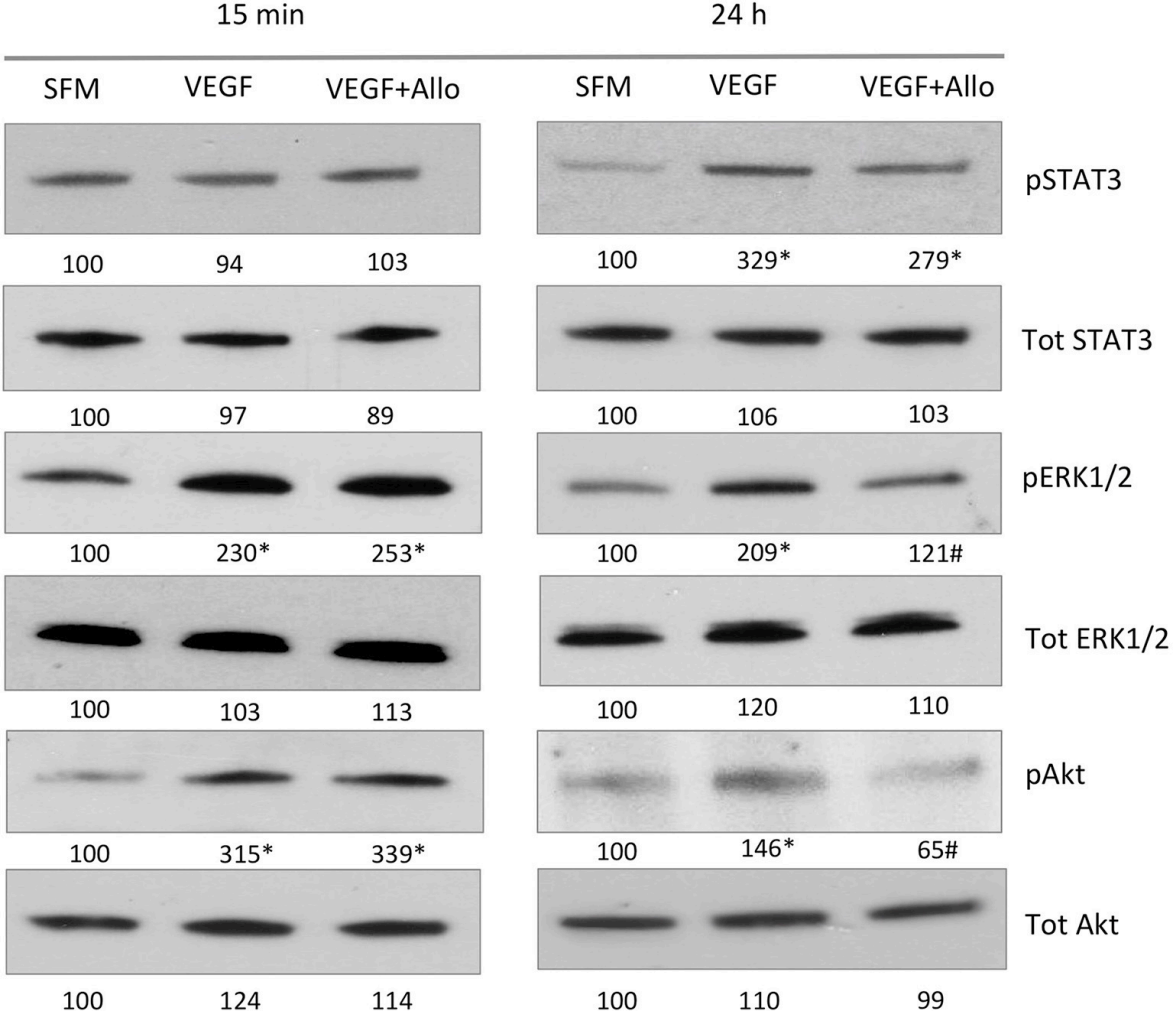
**Allo-aca inhibits several VEGF-induced signaling pathways**. RF/6A cells were stimulated for 15 min or 24 h with 100 ng/mL VEGF in the presence or absence of 250 nmol/L Allo-aca (Allo); control cells were left untreated in SFM. The expression of phosphorylated (p) and total (Tot) proteins was assessed by WB and quantified as described in Materials and Methods. The representative WB results are shown. The numbers under WB panels represent densitometry values (%) of phosphorylated and total proteins in the blot shown, with the value in SFM taken as 100%. The average increase/decrease values from different experiments are given under Results. Statistically significant differences vs. untreated cells are marked with ^*^ and vs. VEGF with #.

To examine if VEGF activates intracellular pathways indirectly through ObR, we stimulated RF/6A cells with 100 ng/mL VEGF in the presence or absence of 250 nmol/L Allo-aca. Both acute (15 min) and long-term (24 h) effects on signaling molecules such as STAT3, ERK1/2, Akt, which are common for VEGFR and ObR were assessed (Figure 3).

At 15 min, VEGF greatly (*p* ≤ 0.05) increased the phosphorylation of ERK1/2 (130 ± 16%) and Akt (181 ± 31%), but had no significant effect on STAT3 (*p* ≥ 0.05). None of these acute VEGF responses were affected by the presence of Allo-aca (*p* ≥ 0.05). In contrast, at 24 h VEGF induced robust and significant STAT3 activation (203 ± 24%) as well as maintained increased phosphorylation of ERK1/2 (109 ± 11%) and Akt (46 ± 5%) In the presence of Allo-aca, the long-term VEGF effects on ERK1/2 and Akt were well suppressed (by 80 ± 12% and 91 ± 10%, respectively), while the phosphorylation of STAT3 was only moderately reduced (by 50 ± 10%) (*p* ≥ 0.05) (Figure 3).

### Allo-aca inhibits ocular neovascularization in the CNV animal model

Allo-aca has previously been shown to reduce leptin-induced angiogenic effects in ocular endothelial cell models *in vitro* (Scolaro et al., 2013; Parrino et al., 2014), however the efficacy of this inhibitor in a relevant *in vivo* model have never been tested. We used the well-recognized rat CNV model (Shah et al., 2015) to induce robust ocular neovascularization and probe the activity of Allo-aca to suppress laser-induced lesions. As described before, leptin mRNA is detectable in CNV-treated rat eyes (Scolaro et al., 2013).

The efficacy of Allo-aca was tested in parallel with the “standard treatment”, i.e., the VEGF Ab.

**Figure 4:**
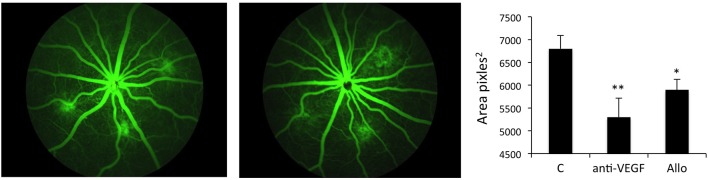
**Allo-aca inhibits laser-induced choroidal neovascularization ***in vivo*****. Choroidal neovascularization (CNV) was induced by bilateral laser treatment as described in Materials and Methods. Three days post-injury, the animals received bilateral intravitreal injections of NaCl, an anti-VEGF antibody 5 μg/eye or Allo-aca 5 μg/eye. Three weeks post-laser treatment, fluorescein angiography was performed and lesion size area was determined with image analysis software. The representative angiography results (fundus images of 3 lesions) in NaCl and Allo-aca treated eyes are shown. The graphs show the average area of lesion size area at the experimental end point. Statistical significance calculated with Student's *t*-test. ^*^p ≤ 0.05; ^**^p ≤ 0.01.

The experiment demonstrated that treatment with either 1 μg anti-VEGF Ab/eye or 5 μg/eye of Allo-aca resulted in significant reduction of lesion size vs. the vehicle control group. When compared with CNV rats injected with NaCl, anti-VEGF intraocular injection inhibited new vessels formation by ~35% (*p* < 0.01) while injections with ObR antagonist reduced neoangiogenesis by ~30% (< 0.05) at 22 days post procedure. Notably, there was no significant difference in lesion size between the group receiving anti-VEGF Ab and Allo-aca (Figure 4). As expected, due to the multifactorial and complex nature of the CNV (Shah et al., 2015), none of the treatments resulted in complete reduction of the lesions.

## Discussion

Several biologic drugs targeting VEGF and/or its receptor have been approved for the use in ophthalmology. At present, VEGF neutralizing drugs, ranibizumab (a humanized monoclonal antibody fragment with molecular weight of 48 kDa), and bevacizumab (a recombinant humanized monoclonal antibody with molecular weight of 149 kDa), that were engineered to bind with high affinity and to neutralize all biologically active isoforms of VEGF (Ferrara et al., 2006; Scolaro et al., 2013) and aflibercept (a fusion protein containing the second immunoglobulin domain of VEGFR-1 and the third immunoglobulin domain of VEGFR-2, which binds all isoforms of VEGF, VEGF-B, and placental growth factor; Holash et al., 2002; Miller et al., 2013; Scolaro et al., 2013) are approved for the treatment of wet AMD and DME, and experimentally used for other eye diseases, e.g., PDR (Willard and Herman, 2012; Scolaro et al., 2013). Most trials have shown benefits with the use of intravitreal anti-VEGF agents for both DME and PDR (Cheung et al., 2010; Scolaro et al., 2013). However, adverse effects (systemic and ocular) and development of resistance to the treatment have been noted with long-term use, thus targeting pro-angiogenic factors other than VEGF could be prove to be an effective alternative or complementary therapy for pathological neovascularization in the eye (Praidou et al., 2010; Tang and Kern, 2011; Truong et al., 2011; Stewart, 2012; Willard and Herman, 2012)

In addition to VEGF, many other molecular players including pro-angiogenic and pro-inflammatory cytokines, anti-angiogenic factors, integrins and matrix proteinases have been implicated in ocular pathologies (Praidou et al., 2010; Truong et al., 2011; Wang et al., 2012). Recent data, including that from our laboratory, suggest that leptin, a cytokine implicated in neovascularization, proliferation and inflammation can induce growth and angiogenic differentiation of ocular endothelial cells *in vitro* (Cao et al., 2001; Suganami et al., 2004; Sun et al., 2011; Scolaro et al., 2013) and might be implicated in DR (Gariano et al., 2000; Parrino et al., 2014). Furthermore, targeting ObR has been shown to inhibit most of biological effects of leptin in ocular disease models (Scolaro et al., 2013; Parrino et al., 2014). This new direction in evaluating leptin a target in ocular neovascularization needs further assessment in light of reports showing differential (and model specific) leptin influence on such components of vasculature as vascular smooth muscle cells (Oda et al., 2001; Bohlen et al., 2007; Rodriguez et al., 2010).

Our previous preliminary observations suggested that simultaneous inhibition of leptin and VEGF pathways was more efficacious in suppressing angiogenesis than individual treatments in HUVEC cells (Ferla et al., 2011). The existence of leptin/VEGF crosstalk has been noted in different experimental models (Sierra-Honigmann et al., 1998; Suganami et al., 2004; Gonzalez-Perez et al., 2010; Ferla et al., 2011; Garonna et al., 2011). However, the functional links between leptin and VEGF have not been sufficiently explored in the context of eye disease and relevant therapeutic interventions. While leptin has been shown to stimulate VEGF expression and potentiate ocular neovascularization, the impact of VEGF on leptin signaling has never been tested.

Here, we demonstrate for the first time that VEGF increases leptin mRNA and protein expression in retinal and corneal endothelial cells. These effects are accompanied by increased cell growth and cell and are abolished in the presence of ObR antagonist, Allo-aca. In addition, VEGF promotes cell chemotaxis and chemokinesis in retinal endothelial cells, which at least in part is restricted by Allo-aca. Likewise, many long-term, but not acute, intracellular signaling effects of VEGF are blocked in the presence of Allo-aca. These VEGF-induced intracellular signals (ERK1/2, Akt) are common for ObR and VEGFR pathways. All these observations suggest that VEGF indirectly activates leptin pathways by upregulating leptin expression, thus, potentiating leptin signaling. Increased leptin concentration, in turn, likely can activate its own expression as well as upregulate VEGF expression, thereby greatly enhancing pro-angiogenic environment in the eye.

We previously demonstrated that inhibition of angiogenesis *in vitro* by combined ObR and VEGF inhibitors is more efficacious than individual treatments (Ferla et al., 2011). Our present study shows that the efficacy of ObR antagonist Allo-aca in restricting experimental neovascularization in animal models is similar to that achieved with compounds targeting VEGF. This suggests that development of therapeutic approaches interfering simultaneously or sequentially with both pathways could be clinically beneficial.

## Author contributions

Conception and design of the work (ES, RC, RF); acquisition, analysis, or interpretation of data (RC, DN, RF, LS, LO, ES); drafting and revising of the manuscript and approval of the final version (ES, RF, LO). All authors have agreed to the content of this manuscript and are accountable for all aspects of the work described.

## Funding

This study was supported in part by a research grant from Novo Nordisk Diabetes Innovation Program to ES.

### Conflict of interest statement

A part of this study was supported by a Novo Nordisk Diabetes Innovation Award to ES. The ObR antagonist described in the paper was co-invented by LO and ES and is covered by the US patent 8778890 “Leptin antagonist and methods of use,” currently licensed to Allysta, Inc. for ophthalmology development. The company did not influence or prescreen data before submission for publication. All the other authors declare that the research was conducted in the absence of any commercial or financial relationships that could be construed as a potential conflict of interest.
