# Corrigendum: Designer Leptin Receptor Antagonist Allo-aca Inhibits VEGF Effects in Ophthalmic Neoangiogenesis Models

**DOI:** 10.3389/fmolb.2016.00075

**Published:** 2016-11-18

**Authors:** Roberta Coroniti, Rafal Farjo, Didier J. Nuno, Laszlo Otvos, Laura Scolaro, Eva Surmacz

**Affiliations:** ^1^Sbarro Institute for Cancer Research and Molecular Medicine, Temple UniversityPhiladelphia, PA, USA; ^2^Department of Biology, Temple UniversityPhiladelphia, PA, USA; ^3^EyeCROOklahoma, OK, USA

**Keywords:** leptin, ObR antagonist, peptide drug, VEGF, ocular neoangiogenesis

In the original article, the name of the author Rafal Farjo was misspelled as Rafal Fario.

The correct spelling appears above. The authors apologize for this error. This does not change the scientific conclusions of the article in any way.

The original article has been updated.

## Conflict of interest statement

The authors declare that the research was conducted in the absence of any commercial or financial relationships that could be construed as a potential conflict of interest.

